# Patient and public involvement in the SPRUCE methodology study investigating electronic patient reported outcomes in oncology clinical trials

**DOI:** 10.1186/s40900-025-00742-y

**Published:** 2025-07-01

**Authors:** Morgaine Stiles, Monisha Dewan, Georgina Manning, Jessica Maudsley, Diana King, Jacqui Gath, Andy Deutsch, Esme Radin, Kim Watson, Franko Kowalczuk, Stephanie Foster, Alexa Gillman, Joanne Haviland, Elizabeth Hill, Lisa Lloyd, Robert Huddart, Emma Hall, Lara Philipps, Rebecca Lewis

**Affiliations:** 1 Clinical Trials and Statistics Unit, The Institute of Cancer Research, London, SW7 3RP UK; 2 Independent Patient Representative, London, UK; 3 Wolfson Institute of Population Health, Queen Mary University of London, London, E1 2AB UK; 4 The Royal Marsden NHS Foundation Trust, Sutton, SM2 5PT UK

**Keywords:** Patient reported outcomes (PROs), Patient reported outcome measures (PROMs), Patient and public involvement (PPI), Electronic patient reported outcomes (ePRO), Study within a trial (SWAT), Digital literacy

## Abstract

**Background:**

Patient reported outcomes (PRO) provide crucial insight into trial participants’ experience of oncology treatments. At the Clinical Trials and Statistics Unit at the Institute of Cancer Research (ICR-CTSU), these are completed by participants on paper. The SPRUCE study within a trial (SWAT) investigates the impact of PRO questionnaire modality (paper or electronic) on the data received. To ensure SPRUCE is acceptable and appropriately patient-focused, we involved Patient and Public Involvement (PPI) partners throughout development and oversight.

**Body:**

A survey was developed with PPI input, to assess public attitudes to electronic completion of healthcare questionnaires. We advertised in local papers to reach respondents without internet access; other avenues were limited due to the COVID-19 pandemic. Survey respondents were invited to virtual discussion groups to review the proposed SWAT design and provide feedback on its relevance and acceptability to potential study participants. Discussion group contributors were invited to join the SPRUCE Patient and Public Oversight Committee, providing PPI input throughout the study. Committee members were given a document explaining clinical trials, the SPRUCE study, and the committee itself. The first committee meeting saw PPI members testing the electronic PRO (ePRO) system and giving feedback on this and the patient-facing documents, for which we provided structured feedback forms. Members also provided feedback on the meeting itself. Of the fifty survey respondents, eight joined a discussion group. Six subsequently joined the Patient and Public Oversight Committee, along with one patient advocate who had been involved in the initial study design and funding application. Each committee member had access to the internet and would prefer to complete PRO questionnaires electronically. Six committee members tested the online ePRO completion system using various personal devices, resulting in changes including the addition of a free text box for participants to leave comments. Patient and public input also shaped patient-facing study documentation, including wording of the patient information sheet and correspondence to participants.

**Conclusions:**

Despite challenges faced in accessing a diverse demographic, PPI input has improved SPRUCE by ensuring the patient viewpoint is central to study oversight, helping identify ways to improve participant experience and streamlining study processes.

**Supplementary Information:**

The online version contains supplementary material available at 10.1186/s40900-025-00742-y.

## Abstract (significance)

People taking part in clinical trials are often asked to complete questionnaires about treatment side-effects. In trials run by our unit, we usually ask people to complete these on paper, however it is now possible for questionnaires to be completed electronically over the internet. We designed a study, SPRUCE, to look at acceptability of electronic questionnaires, and whether they are as likely to be returned electronically as on paper. Patients and members of the public help to make sure research is acceptable and patient-focused. It has been particularly important to involve patient advisors in SPRUCE, because it looks at a new way of collecting information from people. Before starting SPRUCE, we surveyed the public about completing healthcare questionnaires electronically, and their experience of computer technology. Most people who completed our survey were happy to complete questionnaires electronically. We asked people who completed our survey to take part in discussion groups to give feedback on the final design of SPRUCE. A Patient and Public Oversight Committee was formed from people who took part in the discussion groups. The committee helped to test the system for sending electronic questionnaires, gave advice on the information SPRUCE participants would need, and reviewed wording to make sure the electronic system and all documents were easy to use and understand. They also helped develop surveys to find out more about the background of participants and their experience of completing questionnaires, and a letter to update participants when the planned number of people had joined SPRUCE.

**Supplementary Information:**

The online version contains supplementary material available at 10.1186/s40900-025-00742-y.

## Background

The collection of patient reported outcomes (PRO) is critical in clinical trials to assess experience of treatments and interventions from the patient perspective. This enables insight into trial participants’ symptoms without external interpretation from their medical team [1].

At the Clinical Trials and Statistics Unit at the Institute of Cancer Research (ICR-CTSU), PROs are collected on paper, either being sent directly to trial participants’ home addresses, or given out in hospital clinics. However, technology now allows electronic patient reported outcomes (ePRO) collection to be used.

Benefits of ePRO may include increased ease of questionnaire completion, as patients completing questionnaires electronically do not need to attend a clinic or send their completed questionnaires by post. This may lead to more trial participants completing their questionnaires, resulting in increased questionnaire return rates and potentially greater understanding of patients’ symptoms, more robust trial findings, and ultimately improved care for patients. Electronically sending questionnaires directly to participants also decreases administrative burden on hospital sites participating in clinical trials, therefore potentially reducing costs, both financial and resourcing [2].

However, there are barriers to ePRO use and it may not be suitable for all patients. Some may lack access to the necessary technology, or have low levels of confidence or experience using digital technologies [3]. In the UK, 4% of the population are not online, rising to 20% of households comprising only one adult aged 65 years and over, and 25% of the population have low digital capability [4, 5]. Those with fewer digital skills tend to be older and/or less affluent [6], characteristics which correlate with higher cancer incidence [7, 8].

There are limited published data exploring interpatient differences in PRO responses by questionnaire modality. As part of the introduction of ePRO within oncology trials conducted by ICR-CTSU [2], a study within a trial investigating ePRO within oncology clinical trials (SPRUCE) was developed to investigate completion rates and data quality of health-related quality of life ePRO compared to paper PRO [9].

**Fig. 1:**
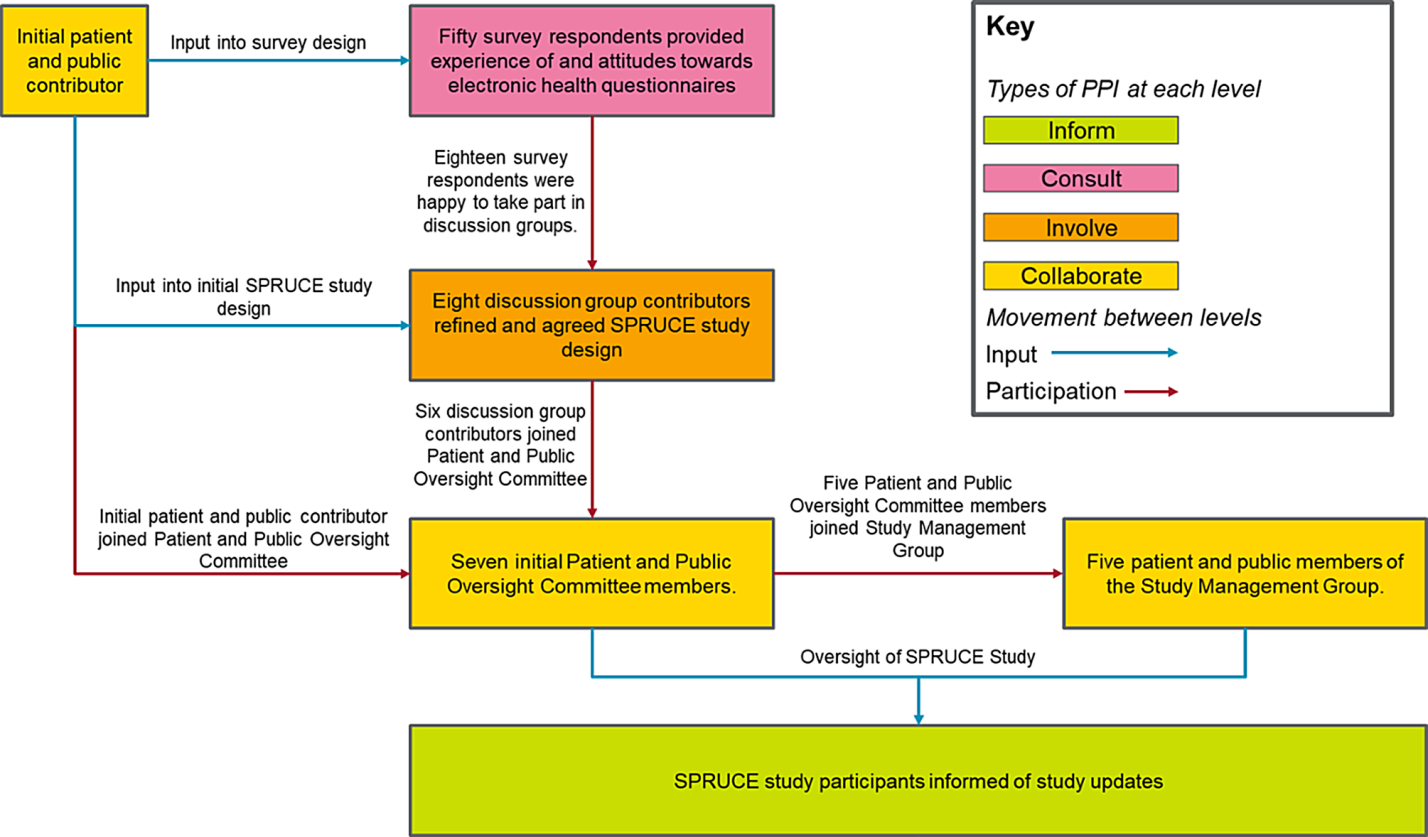
Overview of PPI in the SPRUCE study

At ICR-CTSU we routinely involve patient representatives in trial development and oversight. Because SPRUCE was focused on patient reported outcomes, involving implementation of a new patient-facing data collection system and direct contact with study participants, it was crucial to involve patient and public advisors from the outset. This included ensuring user-centred design principles had been considered by working together to test the system during setup [10]. An overview of the PPI process used to inform the study is illustrated in Fig. 1.

Here, we describe how patient and public involvement was integral to the SPRUCE study, the benefits it brought, and the challenges experienced.

## Public survey on attitudes to electronic healthcare questionnaires

### Survey methods

A public survey, designed in collaboration with a patient advisor, was conducted from February 5th 2021– June 16th 2021 to assess experience of computer technology and attitudes towards electronic completion of health questionnaires (appendix 1). This survey could be completed either electronically or on paper. Those completing the survey were asked about participating in a discussion group to review an electronic system. It was hoped that by running a public survey and inviting all survey respondents to participate in further discussions, the diversity of discussion group contributors would be as representative of the UK public as possible.

**Fig. 2:**
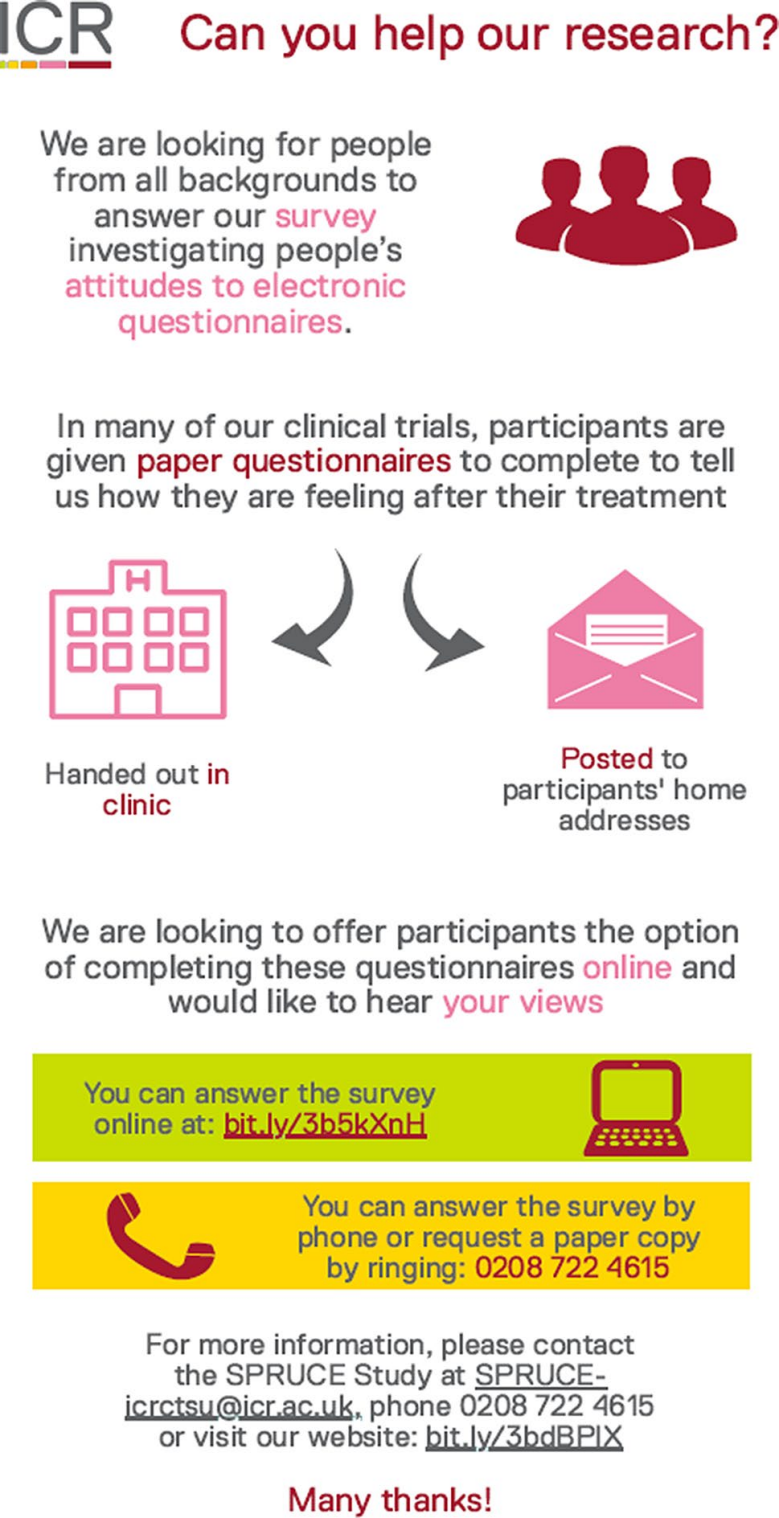
Infographic advert to disseminate survey

An infographic-style advert was designed to help disseminate the survey (Fig. 2). The aim was to reach a wide range of people, including those with limited access to the internet and computer technology. The survey was circulated on the ICR-CTSU’s Twitter account [11], and advertised on the Biomedical Research Centre at the Royal Marsden and the ICR Cancer Patients’ Voice digital platform [12]. In the absence of in-person opportunities as a result of COVID-19 lockdowns, an advert was also circulated to regional University of the Third Age (U3A) groups [13], via a local circular magazine, and distributed to other groups on an ad hoc basis using existing links within the study team. Patients attending a telephone prostate cancer clinic at the Royal Marsden NHS Foundation Trust were invited by their clinician (LP) to complete the survey. Those who were interested in taking part were sent a paper survey with a pre-paid return envelope.

### Survey analysis

Survey responses were analysed descriptively with the percentage of respondents selecting each categorical answer tabulated and displayed graphically. Response rate could not be calculated as the survey was shared in public channels, so the number of people reached is unknown. All responses were of equal interest, and there was no primary outcome measure. The association between respondent demographics and responses to questions regarding the use and experience of technology was reviewed and presented as a percentage of each demographic characteristic category choosing each response. Statistical significance was determined using the Fisher’s exact test with a significance level *p* < 0.05.

### Survey results

Fifty people responded to the survey. Table 1 presents their demographic information. Age range was skewed to people older than 50, with 68% (34/50) 61 years or older. None were younger than 30. 20% (10/50) of respondents’ highest level of education was compulsory school education.

**Table 1:** Survey respondents’ demographics

	*N* = 50			%		
**Method of survey completion**						
Online - BRC Platform	28			56.0		
Online - ICR Surveys	5			10.0		
Paper	17			34.0		
	**Online** ***N*** = **33**		**Paper** ***N*** = **17**		**Total** ***N*** = **50**	
	N	%	N	%	N	%
**Age (years)**						
< 30	0	0.0	0	0.0	0	0.0
31–40	4	12.1	0	0.0	4	8.0
41–50	2	6.1	0	0.0	2	4.0
51–60	9	27.3	1	5.9	10	20.0
61–70	10	30.3	3	17.6	13	26.0
71–80	7	21.2	7	41.2	14	28.0
81–90	1	3.0	6	35.3	7	14.0
> 90	0	0.0	0	0.0	0	0.0
**Gender**						
Female	25	75.8	5	29.4	30	60.0
Male	8	24.2	12	70.6	20	40.0
**Ethnicity**						
Asian/Asian British	0	0.0	1	5.9	1	2.0
White	33	100.0	16	94.1	49	98.0
**Household Income**						
Less than £18,000	4	12.1	3	17.6	7	14.0
£18,001-£25,000	5	15.2	3	17.6	8	16.0
£25,001-£35,000	5	15.2	1	5.9	6	12.0
£35,001-£40,000	1	3.0	2	11.8	3	6.0
£40,001-£50,000	4	12.1	3	17.6	7	14.0
>£50,000	10	30.3	2	11.8	12	24.0
Prefer not to say	4	12.1	3	17.6	7	14.0
**Highest level of education**						
Less than compulsory education (left school prior to age 16)	0	0.0	0	0.0	0	0.0
Compulsory school education (up to the age of 16)	3	9.1	7	41.2	10	20.0
Apprenticeship	0	0.0	4	23.5	4	8.0
Further education (6th form college or equivalent)	7	21.2	2	11.8	9	18.0
Higher Education (undergraduate degree)	13	39.4	2	11.8	15	30.0
Higher Education (post graduate degree)	2	6.1	1	5.9	3	6.0
Professional qualification (e.g. accountancy/nursing)	8	24.2	1	5.9	9	18.0
**Experience of cancer/clinical trials**						
Previous or current participant within a clinical trial	6	18.2	10	58.8	16	32.0
Patient currently receiving cancer treatment (but not participating within a clinical trial)	7	21.2	2	11.8	9	18.0
Patient who has previously received treatment for cancer (but did not participate within a clinical trial)	8	24.2	3	17.6	11	22.0
Healthcare professional/researcher	3	9.1	0	0.0	3	6.0
Other member of the public	9	27.3	2	11.8	11	22.0

Use and experience of people completing electronic questionnaires is shown in Table 2. There was no statistically significant association between internet access and age (*p* = 0.51), although all respondents without internet access were over 70. There was also no statistically significant association between internet access and education level (*p* = 0.10), although more respondents with higher formal education levels had internet access. There was an association between household income and internet access, with all respondents lacking internet access having a household income of less than £18,000 per annum (*p* = 0.01).

**Table 2:** Survey respondents’ use and experience of technology

	*N* = 50	%
Technology respondents are comfortable using *		
Laptop or desktop computer	44	88.0
Smart phone	40	80.0
Tablet computer	38	76.0
None of the above	2	4.0
**Owner of an email address**		
Yes	49	98.0
No	1	2.0
**Access to internet at home or via mobile**		
Yes	47	94.0
No	3	6.0
**Previous experience completing an online survey**		
Yes	42	84.0
No	8	16.0
**Previous use of an online reporting tool in relation to health**		
Yes	24	48.0
No	26	52.0

*participants can select more than one method, percentages are out of total participants

84% (42/50) of respondents had previously completed an online survey, and 48% (24/50) had used an online reporting tool in relation to their health. Despite a very high percentage (94%, 47/50) of respondents having internet access, only 76% (39/50) of survey respondents said they would prefer to complete a PRO questionnaire electronically (Table 3).

**Table 3:** Survey respondents’ healthcare questionnaire preferences

	*N* = 50	%
**Would you prefer to answer a health questionnaire on paper or online (via a website or mobile phone application)?**		
**Online**	38	76.0
***If you were answering a healthcare questionnaire online would it be more comfortable using a website or a mobile phone application?***		
*Website*	*24*	*63.2*
*Mobile phone application*	*4*	*10.5*
*No preference*	*10*	*26.3*
***If you were to complete the health questionnaires electronically would you be happy to receive emails***,*** or notifications on your phone***,*** to let you know you have questionnaires available to complete?***		
*Yes*	*34*	*89.5*
*No*	*4*	*10.5*
**Paper**	11	22.0
***If your preference would be paper: Would you be willing to complete a health questionnaire online if requested?***		
*Yes*	*6*	*54.5*
*No*	*4*	*36.4*
*N/A*	*1*	*9.1*
***If you were answering a healthcare questionnaire online would it be more comfortable using a website or a mobile phone application?***		
*Website*	*7*	*63.6*
*Mobile phone application*	*1*	*9.1*
*No preference*	*1*	*9.1*
*Missing*	*2*	*18.2*
***If you were to complete the health questionnaires electronically would you be happy to receive emails***,*** or notifications on your phone***,*** to let you know you have questionnaires available to complete?***		
*Yes*	*6*	*54.5*
*No*	*3*	*27.3*
*Missing*	*2*	*18.2*
**Paper/Online (ticked both)**	1	2.0
	*N* = 50	**%**
**I am worried about potential breaches in the privacy of my data when completing a health questionnaire online**		
Agree	8	16.0
Neither agree or disagree	15	30.0
Disagree	18	36.0
Strongly disagree	8	16.0
Missing	1	2.0
Median (IQR)*	2	(2,2)
**I am worried about potential breaches in the privacy of my data when completing a health questionnaire on paper and sending it in the post**		
Agree	10	20.0
Neither agree or disagree	15	30.0
Disagree	20	40.0
Strongly disagree	5	10.0
Median (IQR)*	2.5	(2,3)
**I am worried about potential breaches in the privacy of my data when completing a health questionnaire in a clinic**		
Agree	4	8.0
Neither agree or disagree	15	30.0
Disagree	19	38.0
Strongly disagree	10	20.0
Missing	2	4.0
Median (IQR)*	2	(2,3)

* where higher values indicate stronger agreement (Strongly agree = 5, Agree = 4, Neither agree or disagree = 3, Disagree = 2, Strongly disagree = 1)

**Fig. 3:**
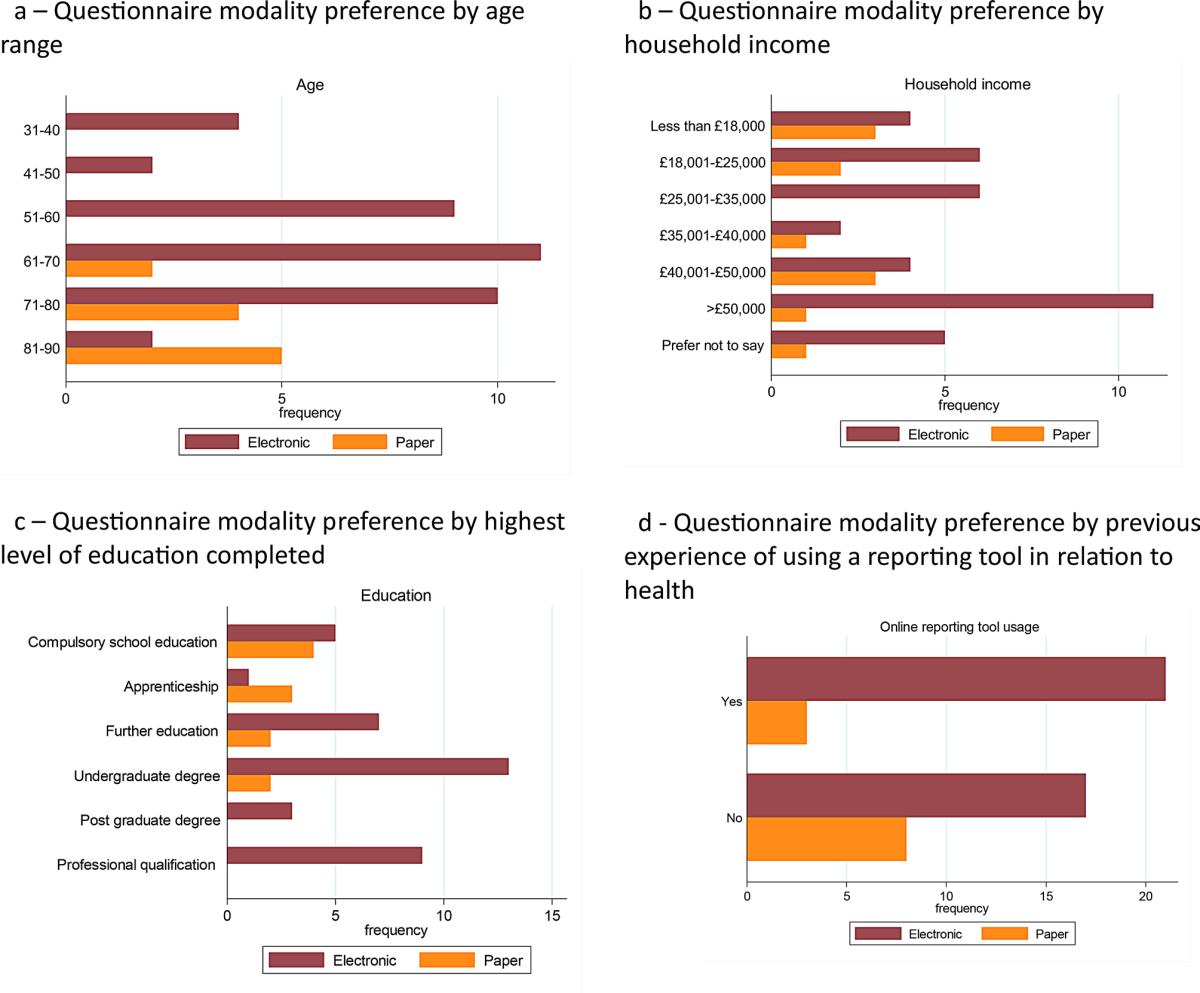
Results from survey assessing public attitudes to completing healthcare questionnaires online. **a** Questionnaire modality preference by age range **b** Questionnaire modality preference by household income **c** Questionnaire modality preference by highest level of education completed **d** Questionnaire modality preference by previous experience of using a reporting tool in relation to health

The results suggested that only respondents aged 61 or over preferred paper questionnaires (*p* = 0.02) (see Fig. 3a). Figure 3b illustrates that there was no significant association between preferred modality and income (*p* = 0.29). However, those with higher levels of education were more likely to prefer electronic questionnaires (*p* = 0.03), as shown in Fig. 3c. There was no significant association between respondents having prior experience of using an online reporting tool in relation to their health and modality (*p* = 0.17) (see Fig. 3d). Of those respondents with a preference for paper, 58% (7/12) were willing to complete questionnaires online if requested.

Eighteen of the 50 people (36%) who completed the survey were happy to be contacted regarding the discussion group.

### Survey findings - summary

The survey reached people in older age groups, more likely to be diagnosed with cancers studied at the ICR-CTSU than those of a younger demographic. Respondents had a range of different incomes, and many had experience of cancer treatment, either within or outside of a clinical trial. The ethnic diversity of survey respondents was substantially lower than that of the overall UK population (98% White vs. 82% White in 2021 England and Wales Census) [14]. It was challenging to reach people who were not online, exacerbated by restrictions imposed by the COVID-19 pandemic.

Despite the high percentage of respondents who had internet access, a quarter preferred to complete PRO questionnaires on paper, indicating that technology access is not the only factor dictating preference. Concerns about confidentiality were similar across electronic and paper methods, suggesting that security considerations do not influence format preference.

## PPI discussion groups

The initial SPRUCE design was developed in collaboration with a patient advisor. To gain wider feedback on acceptability and refine the study design, we held discussion groups with patients and members of the public. Discussion group contributors expressed their interest in participation via the survey described above.

### Discussion group methods

Eight of the 18 survey respondents (44%) who expressed interest in discussion groups participation attended a session.

Two discussion group sessions were held on 8th September 2021 and 20th September 2021. These were led by LP, and consisted of short sections of presentation, followed by questions designed to elicit a conversation around the topic. Topics for the discussion group focused on barriers to electronic questionnaire completion and ways to mitigate these, the proposed study design and endpoints. Facilitating researchers (MS, GM) took written notes of discussions.

Prior to the discussion groups contributors were provided with an optional pre-read document which provided background information on ICR-CTSU, clinical trials and methodology research.

Meetings were held over Zoom due to restrictions imposed by the COVID-19 pandemic. Tablets were available for contributors without internet access to support their attendance. Contributors were reimbursed for their time in line with national guidelines, supported by a grant from the National Institute for Health and Care Research (NIHR) Biomedical Research Centre at The Royal Marsden NHS Foundation Trust and The Institute of Cancer Research, London (B111). During the discussion group and in all further Patient and Public Involvement (PPI) activities within the study, effort was made to ensure that researchers did not outnumber patient advocates, to facilitate and enable discussion.

### Discussion group outcomes

**Fig. 4:**
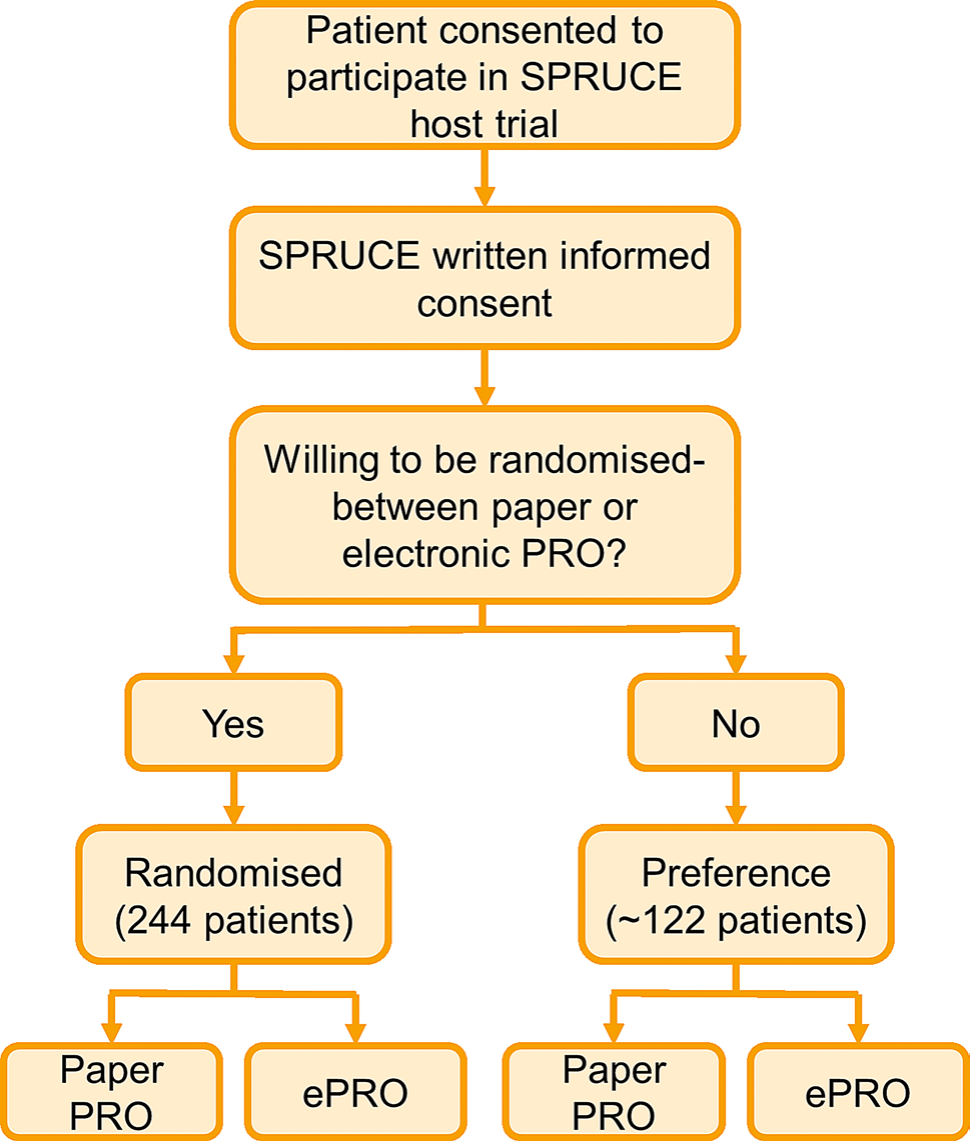
SPRUCE study design

**Fig. 5:**
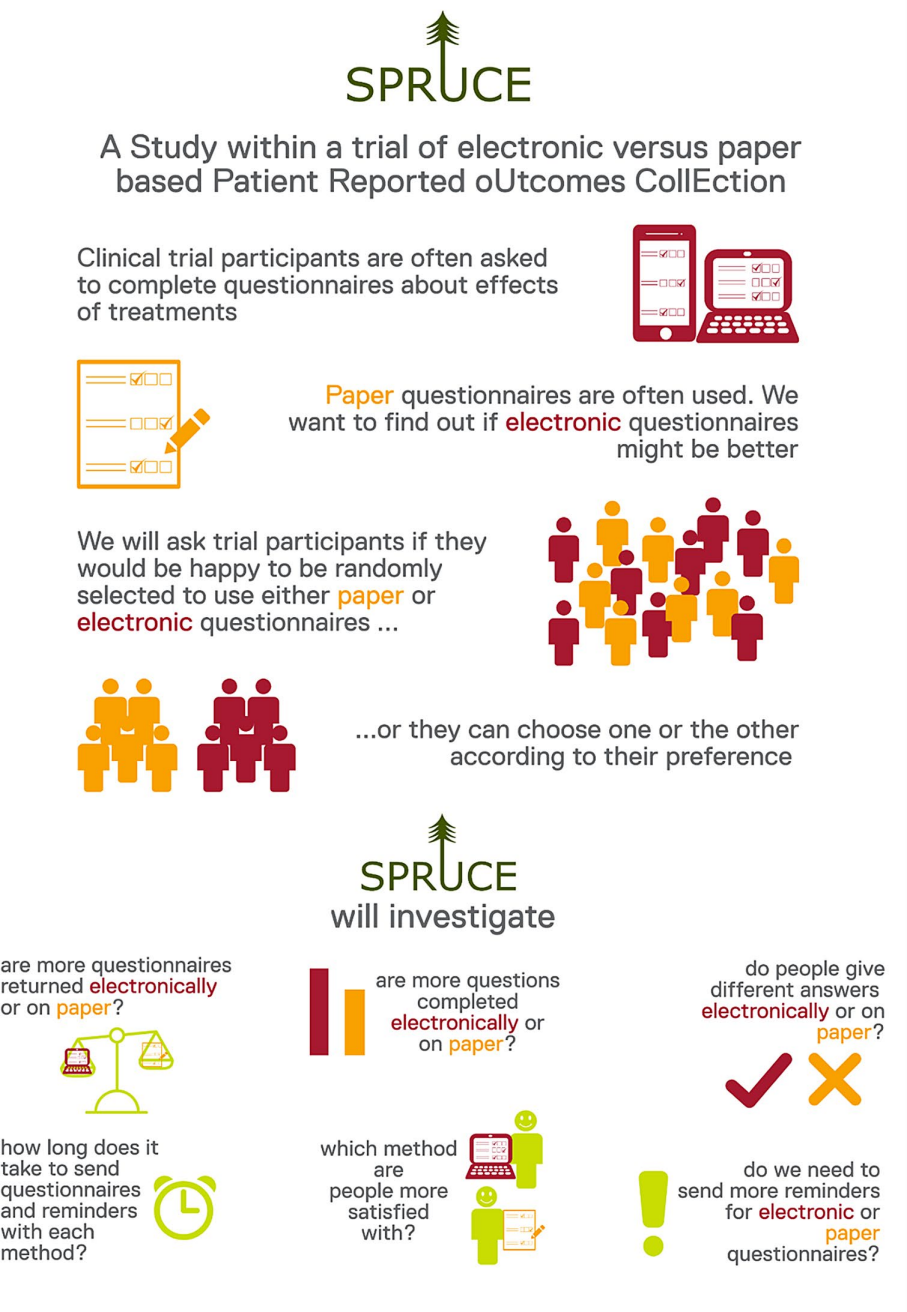
SPRUCE study infographic

Figures 4 and 5 show the agreed study design and accompanying infographic. SPRUCE participants would be asked whether they would accept randomisation 1:1 between paper or electronic questionnaire completion. Under the partially randomised design, participants could select their preference of paper or electronic questionnaires if randomisation was not acceptable. The SPRUCE study was designed to run across existing ICR-CTSU host trials, with the aim of assessing whether ePRO response rates were similar to ICR-CTSU paper PRO response rates.

Discussion group contributors advised those completing questionnaires electronically should be able to save their responses and return to them, rather than having to complete questionnaires in one sitting. They should also be able to navigate backwards through a questionnaire to see previous answers. Study participants should receive information about who to contact if they had a question regarding the study or the questionnaires. The importance of continued communication with study participants was emphasised, including providing study results when available. Discussions also highlighted the importance of monitoring recruitment into the host trials to ensure that there was no impact of SPRUCE on trial enrolment. Table 4 summarises all the suggestions made within the discussion groups by theme.

**Table 4:** Patient advocate suggestions from the PPI discussion groups

Suggestion	Implementation
***Participant information and support***	
Provide reassurance to participants that their data is safe when they are completing it electronically.	Implemented
Provide participants with feedback on the trial and trial results.	Implemented
Provide the option for a carer or relative to help the participants to complete the questionnaire electronically.	Not implemented as PRO questionnaires are designed to be completed by the participant themselves
Provide participants with instructions on how to complete the questionnaires electronically.	Implemented
Provide clear contact details for if a participant has an issue.	Implemented
***System set-up***	
Allow participants to easily navigate backwards and forwards through the questionnaire to go back and see or change answers before submitting the questionnaire.	Implemented
Provide a summary of the participants’ answers at the end of the questionnaire so they can see their answers and edit them if needed before submission.	Not implemented due to system restrictions
Allow questionnaires to be saved so participants can return to their questionnaire at a later time if required.	Implemented
Allow participants to continue completing questionnaires electronically at the end of their time in SPRUCE.	Implemented
Consider calling participants on the telephone as a reminder if they do not complete the questionnaire.	Not implemented due to resource requirements– participants are sent postal or email reminders instead
***Host trial considerations***	
Monitor recruitment into the host trials to ensure that the inclusion of SPRUCE does not impact their enrolment.	Implemented

Although tablets were available for those unable to join discussion groups remotely, all contributors had internet access, so this was not required.

### Discussion group summary

The lack of ethnic diversity amongst survey respondents was reflected in discussion group contributors, a limitation of the process of using survey responses to identify contributors. The main concern expressed with the study design was that the potential existed for all participants to have a strong preference and refuse randomisation. A feasibility of randomisation stage was therefore included, and the final protocol included the option of switching to a patient preference design should sufficient randomisations not be observed within the first 50 patients that entered SPRUCE. Randomisation would not be considered feasible if more than 50% of participants declined randomisation or subsequently declined their allocated modality. Overall, the discussion group contributors supported the objectives of SPRUCE and agreed that the proposed study design was acceptable.

## Patient and public oversight committee

After the discussion groups, we constituted the SPRUCE Patient and Public Oversight Committee to provide PPI advice throughout the study’s duration. All discussion group contributors were invited to join. Six contributors and the patient advocate involved with the initial study design agreed to join. Five of the seven independent patient and public members of the group had existing experience of patient advocacy, and all but one of the independent members had personal experience of cancer. Five of the independent Patient and Public Oversight Committee members also sat on the Study Management Group, providing additional input as part of a multidisciplinary group of site staff, trialists and patient advocates.

### First patient and public oversight committee meeting

The first meeting was held on 2nd February 2022. Prior to the meeting, members signed a charter which set out the roles and responsibilities of committee members and ICR-CTSU. The committee could be chaired by either a patient advocate member or a member of the ICR-CTSU. It was agreed that meetings would be held online every six months and papers would be sent to members by email, with the option of receiving hard copies in the post if preferred.

The first meeting reviewed the ePRO system and participant-facing materials. Members volunteered to test the system after the meeting, providing further structured feedback captured on a bespoke form. This included the type of device and browser used and captured opinions on instructional text, usability, suggested improvements, and any issues encountered. Members were also asked to complete a meeting and paperwork form to provide feedback on the study documentation and meeting itself. We asked for opinions on the structure and contents of the meeting and provide suggestions for future meetings, to ensure that everyone was sufficiently supported and future meetings could be adapted as needed.

### First patient and public oversight committee meeting recommendations

Recommendations from the committee regarding the ePRO system, participant-facing documents and future meetings (see Table 5) were implemented where possible. However, some could not be carried out due to limitations within the ePRO system, or due to the need for consistency between the questionnaires within SPRUCE and those in the host trials.

**Table 5:** Patient advocate suggestions from the first patient and public oversight committee meeting

Suggestion	Implementation
***Participant information and support***	
Set up a helpline to call if participants had any issues.	Implemented
Add an example of a completed paper and electronic questionnaire question into the participant information sheet.	Implemented
Add an infographic detailing the SPRUCE study into the participant information sheet.	Implemented
Provide information on the devices that can be used to complete the ePRO questionnaires.	Implemented
Collect the demographics of study participants to assess whether there is a difference in demographics between people who agree to be randomised and those who choose to complete questionnaires electronically or on paper.	Implemented– demographics form created based on this
***System set-up***	
Provide a N/A or prefer not to answer button for all the questions in the questionnaire.	Not implemented as this would invalidate the questionnaires and not align with the host trial questionnaires.
Add explanatory text to the database, including required date format.	Implemented
Add a note asking participants to check over their answers at the end of the ePRO survey.	Implemented
Provide clear instructions on how to resize their browser if the participant experiences display issues.	Implemented
Add a message to the system to thank participants for agreeing to take part in the study.	Implemented
Add a bar to show participants how far through the questionnaire they are.	Not implemented due to system constraints
Add a sentence that says ‘Once you press the Submit button, you will no longer be able to return to the survey.’	Partially implemented - wording changed further following feedback from patient advocate members of the Oversight Committee
Add contact details to the system that participants can use if they encounter any technical issues.	Implemented
Add an alert if any questions are missed before submission of the questionnaires, in case they are missed in error.	Not implemented due to system constraints
Test the ePRO system’s useability with accessibility add-ons.	Implemented
Set up both an email link to the surveys, and a login portal system.	Not implemented due to system constraints
Add a reminder to the thank you email upon submitting an ePRO questionnaire that the participant’s answers will be used in their host trial as well as SPRUCE.	Implemented
***Patient and Public Oversight Committee study updates***	
Send regular updates between meetings about study progress.	Implemented
Include a comparison of enrolment into the host trials against the enrolment into SPRUCE in future meeting reports.	Implemented
Provide feedback from centres and participants where available.	Implemented

Members suggested that differences between ePRO and paper PRO uptake, adherence and acceptability should be explored in different groups of people. This led to a survey being developed in collaboration with committee members to capture SPRUCE participants’ demographics, and the addition of a secondary endpoint to assess differences in demographics between the patient preference groups.

**Fig. 6:**
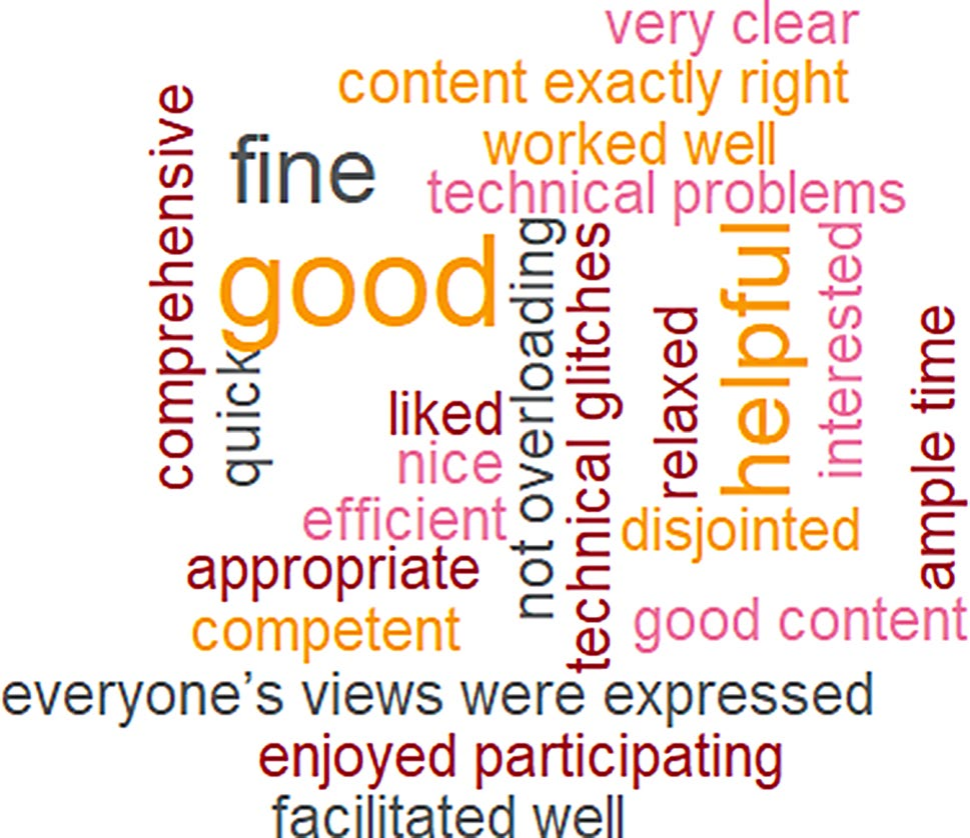
Feedback from patient advocates on the first Patient and Public Oversight Committee meeting

Patient advocates requested information for review at future meetings, incorporated into the reports provided. Further comments regarding the first meeting are summarised in Fig. 6. Feedback was largely positive. It was noted that the dynamic between the independent members was expected to improve over time as members got to know each other. This emphasises the importance of researchers in the group acting as effective mediators of discussions, ensuring everyone has sufficient time and opportunity to contribute. There were also some technical issues with the meeting, due to holding it online, which were resolved in future meetings.

### Subsequent patient and public oversight committee meetings

Subsequent meetings have discussed study progress, including any arising challenges, with review of any patient-facing documentation requiring changes. A report is circulated prior to, and discussed within, the meeting, providing information regarding feedback from the Study Management Group, centre and host trial set-up status, enrolment figures, updates regarding PRO booklet return rate, a summary of participant demographics, a summary of planned and approved amendments, and a summary of presentations and publications. After each meeting, minutes from the meeting are circulated, and details of how to claim reimbursement are provided. This is given as per the Cancer Research UK guidelines [15].

Members are sent monthly email updates between meetings regarding enrolment figures, site set up progress and any other important information pertaining to the study.

Throughout the study, patient advocate members have provided guidance on all SPRUCE-related matters, from providing continued input on updated study documentation, to advising on the dissemination of study results and reviewing and co-authoring abstracts and publications.

### Patient and public oversight committee– summary

While all ICR-CTSU trials have some level of PPI input, having a dedicated Patient and Public Oversight Committee is unusual, and provided an additional level of PPI input into SPRUCE. The method of asking survey respondents to participate in a discussion group, and discussion group contributors to participate in the Patient and Public Oversight Committee has meant that the study has an oversight committee of people who are interested in and informed about the study throughout.

However, the limitations in diversity have been propagated throughout the process, from the survey not reaching a diverse group of people leading to the Patient and Public Oversight Committee having limited diversity. Of particular note is the fact that no members of the Patient and Public Oversight Committee indicated that they would prefer to complete a questionnaire on paper rather than online, meaning that the views of the group are skewed towards ePRO.

It is vital to ensure that the introduction of ePRO considers the needs of trial participants. Some feedback from our patient advocates could not be implemented in SPRUCE due to constraints either from the system or needs of the host trial. For example, suggested changes to validated questionnaires would render responses unusable in the analysis of the host trial PRO studies. The use of forms to capture feedback was helpful to allow patient advocate committee members the time to make a detailed assessment of the system and documentation, and structure their feedback.

## Discussion and limitations

The PPI discussion groups highlighted the importance of creating a user-friendly ePRO system that could be used by individuals of all ages and reflected the ease and simplicity of completing a paper questionnaire. Clear instructions on how to complete the questionnaire electronically were implemented, as well as contact details should the participant require help. It was agreed to provide reassurance that participants’ data would be kept safe to mitigate scepticism around providing data online. Allowing individuals to navigate back and forth through the questionnaire and pause the questionnaire to complete later was important to allow flexibility for completing the questionnaire with no time constraints and view the questionnaire in its entirety before completing. This would allow people completing the questionnaire to view and change their answers as per paper questionnaires, relieve time pressures and prevent digital fatigue.

Although the contribution of our patient and public advisors has been invaluable throughout this project, it was challenging to identify and engage with a diverse range of people representative of the UK public as a whole. We suggest researchers undertaking a similar process in future reach out to a variety of community groups with the aim of increasing the diversity of people reached.

Despite attempts to ensure that people with lower levels of digital literacy could be involved, this was greatly hampered by the COVID-19 pandemic meaning that traditional routes to engage potential survey respondents face to face were not possible. In addition, many of the patient advocates participating in the discussion groups and Patient and Public Oversight Committee were already involved in patient advocacy. It was extremely helpful to have experienced PPI advisors, particularly for this methodology study where a level of existing knowledge and experience is useful to help navigate discussions. However, it was hoped that by inviting survey respondents to have further involvement with the project, people new to patient advocacy would be able to provide a new, outside perspective on the study.

## Conclusions

Overall, we believe that the approach we took to embedding PPI within SPRUCE has been extremely worthwhile and highly beneficial to the study. Feedback from the discussion groups ensured that the study design was appropriate and patient-centric, patient advocate testing of the database ensured that this was user friendly, and input into the patient facing documentation meant that these documents were more accessible for study participants. With any PPI activity, and especially in a study where patient advocates hold such a central position, it was important that a good relationship was maintained with the patient advocates, and that they were sent regular updates regarding study progress. The iterative approach to PPI taken in SPRUCE, where patient and public advocates involved in early exploratory stages of study development were invited to later oversight stages, aided development of relationships between our patient partners and the study team. A dedicated Patient and Public Oversight Committee, in addition to having patient partner members of the Trial Management Group, was invaluable for ensuring a greater depth of PPI input into the study. In a methodological study with intensive participant contact, ensuring that patient advocates have oversight at the same level as collaborating health professionals is highly recommended.

## Electronic supplementary material

Below is the link to the electronic supplementary material.

Supplementary Material 1

Supplementary Material 2

## Data Availability

Data is provided within the manuscript, figures, tables and supplementary information.

## Abbreviations

PRO: Patient Reported Outcomes
SWAT: Study Within A Trial
ePRO: Electronic Patient Reported Outcomes
PPI: Patient and Public Involvement
ICR-CTSU: Institute of Cancer Research Clinical Trials and Statistics Unit
SPRUCE: A Study within a trial of electronic versus paper based Patient Reported oUtcomes CollEction
U3A: University of the Third Age
NIHR: The National Institute for Health and Care Research
