# Supplementary material for: Patient and public involvement in the SPRUCE methodology study investigating electronic patient reported outcomes in oncology clinical trials
Source: Res Involv Engagem. 2025 Jul 1;11:70. doi: 10.1186/s40900-025-00742-y (PMC12211921; doi:10.1186/s40900-025-00742-y)
Supplement: Supplementary file 1 — Supplementary Material 1 [file 40900_2025_742_MOESM1_ESM.pdf]

**Attitudes to ePRO PPI survey**

Within health care and clinical trials, questionnaires can be used to collect information from patients on the impact that treatments and health conditions may be having upon their quality of life. This data is called patient reported outcomes (PRO). This can include general information such as mobility and tiredness, as well as more disease or treatment specific questions such as those about bowel movements or sexual function.

Traditionally, these questionnaires are provided to patients on paper for them to complete either during clinic or at home. If these are used as part of a clinical trial, they will then be posted to the research team running the clinical trial for the information to be added to a database.

More recently patients have been offered the opportunity to complete these questionnaires electronically such as via a website or a mobile phone app. Some studies have shown that there can be different factors which can limit an individual's willingness to complete these questionnaires electronically.

The Institute of Cancer Research Clinical Trials and Statistics Unit would like to introduce electronic questionnaires to the participants of the clinical trials it conducts. To be sure that this will be successful and user friendly we would like to gather your opinions about the electronic collection of health questionnaire responses and what may be barriers for trial participants wishing to complete questionnaires electronically.

Your response will be anonymous unless you choose to provide us with your personal details at the end of the survey.

| Individual Details |
|---|
| We are collecting these details in order to understand what groups of participants have filled out the survey so that we can be sure that we are getting a variety of groups of people responding and make sure our results are not biased. |
| **Age**<br>☐ <20<br>☐ 21-30<br>☐ 31-40<br>☐ 41-50<br>☐ 51-60<br>☐ 61-70<br>☐ 71-80<br>☐ 81-90<br>☐ >90 |
| **Gender**<br>☐ Male<br>☐ Female<br>☐ Other |
| **Ethnicity**<br>Please choose one option that best describes your ethnic group or background<br>White<br>☐ English/Welsh/Scottish/Northern Irish/British |

- ☐ Irish
- ☐ Gypsy or Irish Traveller
- ☐ Any other White background, please describe

Mixed/Multiple ethnic groups

- ☐ White and Black Caribbean
- ☐ White and Black African
- ☐ White and Asian
- ☐ Any other Mixed/Multiple ethnic background, please describe

Asian/Asian British

- ☐ Indian
- ☐ Pakistani
- ☐ Bangladeshi
- ☐ Chinese
- ☐ Any other Asian background, please describe

Black/ African/Caribbean/Black British

- ☐ African
- ☐ Caribbean
- ☐ Any other Black/African/Caribbean background, please describe

Other ethnic group

- ☐ Arab
- ☐ Any other ethnic group, please describe

**Household Income**

Approximately what is your household's current total gross/pre-tax salary or income (including pension)?

- ☐ Less than £18,000
- ☐ £18,001-£25,000
- ☐ £25,001-£35,000
- ☐ £35,001-£40,000
- ☐ £40,001-£50,000
- ☐ >£50,000
- ☐ Prefer not to say

**Education**

Which of the following best describes your highest level of education completed?

- ☐ Less than compulsory education (left school prior to age 16)
- ☐ Compulsory school education (up to the age of 16)

- ☐ Apprenticeship
- ☐ Further education ($6^{th}$ form college or equivalent)
- ☐ Higher education (undergraduate degree)
- ☐ Higher education (post graduate degree)
- ☐ Professional qualification (e.g. accountancy/nursing)
- ☐ Other, please specify

**Which of the following best describes you? (please select the most relevant option)**

- ☐ Previous or current participant within a clinical trial
- ☐ Patient currently receiving cancer treatment (but not participating within a clinical trial)
- ☐ Patient who has previously received treatment for cancer (but did not participate within a clinical trial)
- ☐ Healthcare professional/researcher
- ☐ Other member of the public

**Please let us know where you heard about this survey:**

## Your use and experience of technology

**Are you comfortable using any of the following? (please tick all that apply)**

- ☐ Smart phone
- ☐ Tablet computer
- ☐ Laptop or desktop computer
- ☐ None of the above

**Do you currently have an email address?**

- ☐ Yes
- ☐ No

**Do you have access to internet at home or via your mobile?**

- ☐ Yes
- ☐ No

**Have you ever filled in an online survey?**

- ☐ Yes
- ☐ No

**Have you ever used an online reporting tool in relation to your health?**

- ☐ Yes

- ☐ No

**How would you answer a health questionnaire?**

**Would you prefer to answer a health questionnaire on paper or online (via a website or mobile phone application)?**

- ☐ Paper
- ☐ Online

**If your preference would be paper: Would you be willing to complete a health questionnaire online if requested?**

- ☐ Yes
- ☐ No
- ☐ N/A

**If you were answering a healthcare questionnaire online would it be more comfortable using a website or a mobile phone application?**

- ☐ Website
- ☐ Mobile phone application
- ☐ No preference

**If you were to complete the health questionnaires electronically would you be happy to receive emails, or notifications on your phone, to let you know you have questionnaires available to complete?**

- ☐ Yes
- ☐ No

**Please select the most appropriate response for each statement below**

I am worried about potential breaches in the privacy of my data when completing a health questionnaire ***online***

| Strongly disagree | Disagree | Neither agree or disagree | Agree | Strongly agree |
|---|---|---|---|---|

I am worried about potential breaches in the privacy of my data when completing a health questionnaire ***on paper and sending it in the post***

| Strongly disagree | Disagree | Neither agree or disagree | Agree | Strongly agree |
|---|---|---|---|---|

I am worried about potential breaches in the privacy of my data when completing a health questionnaire ***in a clinic***

| Strongly disagree | Disagree | Neither agree or disagree | Agree | Strongly agree |
|---|---|---|---|---|

**Do you have any other thoughts about introducing electronic patient questionnaires into a clinical trial?**

**Focus Group**

**Would you be interested in taking part in a focus group to comment on the design and ease of use of an electronic patient reporting system?**

- ☐ Yes
- ☐ No

If yes please give your name and contact details below,

Name
Address
Telephone number
Email address
