# Supplementary material for: Patient and public involvement in the SPRUCE methodology study investigating electronic patient reported outcomes in oncology clinical trials
Source: Res Involv Engagem. 2025 Jul 1;11:70. doi: 10.1186/s40900-025-00742-y (PMC12211921; doi:10.1186/s40900-025-00742-y)
Supplement: Supplementary file 2 — Supplementary Material 2 [file 40900_2025_742_MOESM2_ESM.pdf]

SPRUCE focus group background information sheet

1 Introduction

Thank you for considering our invitation take part in a focus group looking at the use of electronic patient reported outcomes in clinical trials managed by the Institute of Cancer Research Clinical Trials and Statistics Unit (ICR-CTSU). This pack tells you more about how it will work and some background on the Institute of Cancer Research Clinical Trials and Statistics Unit.

1.1 About the Institute of Cancer Research

The Institute of Cancer Research (ICR) is an independent college of the University of London and is one of the world's most influential cancer research organisations dating back more than 100 years. The ICR works closely with our partner hospital, The Royal Marsden NHS Foundation Trust, and together we form the largest comprehensive cancer centre in Europe, employing several thousand staff. The joint organisation is at the forefront of international cancer research and together we are rated in the top four cancer centres globally.

Further information on the ICR can be found at the following locations:
Website: http://www.icr.ac.uk
Twitter: @ICR_London
Facebook: http://www.facebook.com/theinstituteofcancerresearch

1.2 About the Institute of Cancer Research Clinical Trials and Statistics Unit (ICR-CTSU)

ICR-CTSU designs and manages many national and several international cancer clinical trials, with a focus on:

- Breast cancer
- Urological cancers (includes cancers of the male and female urinary system and the male reproductive organs)
- Head and neck cancers

We also have an expanding portfolio of trials in rarer cancer types including melanoma (usually but not always a cancer of the skin), sarcoma (can affect any part of the body) and also in ovarian and lung cancer. We are based in Sutton, Surrey and employ more than 90 staff, including Statisticians, Trial Managers, Data Managers, IT and administrative support staff.

2 What are clinical trials?

Research studies involving people are called clinical trials. Clinical trials investigate whether a medical treatment, device or even a strategy:

- is safe and effective for use;
- has side effects (whether short or long term);
- works better than the current available approach;
- can improve people's quality of life.

Some trials may also explore which treatments or procedures work best for certain illnesses or groups of people.

Trials are also used to find out the best ways of using treatments, such as:

- the length of time they are given to the patient;
- the dose of a treatment;
- whether they should be used together in combination with other treatments.

A well managed clinical trial is the most reliable way to assess whether or not a treatment is safe and effective. A new treatment is not always better, and can sometimes be less effective than, or not as safe as, the standard treatments currently used. Therefore it is important that new treatments are properly tested in a clinical trial before they are used in standard clinical care.

We need clinical trials in order to improve the treatment and care of patients both now and in the future. Many of the treatments now commonly used in the NHS have been tested in clinical trials. For example, in cancer care, trials have been used to try out new treatments such as radiotherapy, chemotherapy, and surgery. This has meant that many people with cancer and many other illnesses now live longer and have a better quality of life.

More information about the different types and phases of trials can also be found on Cancer Research UK's website at the following location: http://www.cancerresearchuk.org/about-cancer/find-a-clinical-trial.

2.1 How are the rights, safety and well-being of the participants protected in clinical trials?

In order to protect the rights, safety and well-being of the participants and to ensure reliable results clinical trials follow strict scientific and ethical standards. These standards are called Good Clinical Practice (GCP). GCP is a set of internationally-recognised ethical and scientific quality principles that must be followed when designing, conducting, recording and reporting clinical trials that involve people. More information on good clinical practice can be found on: https://www.gov.uk/guidance/good-clinical-practice-for-clinical-trials.

2.2 How are clinical trials developed and organised?

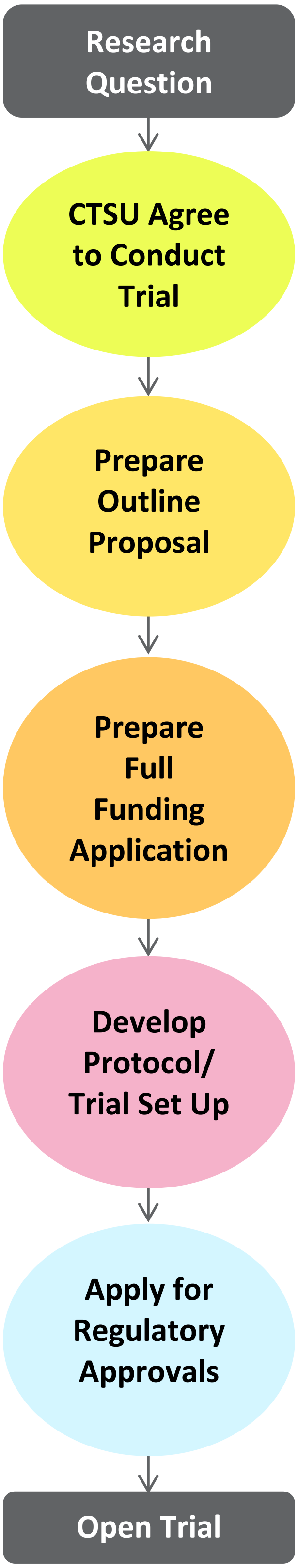

The diagram on the left shows the main stages in the development of a clinical trial. Once a group of researchers has an idea for a trial, they need to develop a research proposal and to secure funding to run the trial. The trial will be led by a Chief Investigator who has overall responsibility for the design, conduct and reporting of the trial. In cancer clinical trials the Chief Investigator will be a registered doctor with an expert knowledge and a strong interest in the area of research of the trial. The ICR-CTSU works closely with the Chief Investigator to submit the funding applications to research charities. These are usually in two stages submitted over approximately one year. Initially an outline application is usually submitted and if successful a more detailed application will follow. Funding may also be obtained from a pharmaceutical company either to add additional support to that obtained from a research charity or in some cases all the funding may come from this source.

If the funding application is successful the researchers need to develop a trial protocol. The protocol describes how a clinical trial will be conducted and ensures the safety of the trial patients and the reliability of the data collected. It is developed by ICR-CTSU and a team of people with expertise in the area being researched, such as doctors, researchers and statisticians. Together they form the Protocol Development Group or PDG. Some trials may also have one or more patient advocate members in the PDG.

The protocol will include:

- why the trial should be done;
- the numbers of patients that are needed (the sample size);
- who can take part in the trial (eligibility);
- detailed information about the treatment plan;
- what tests the patients will have and how often;
- details about how, when and what information (data) will be collected to monitor the health of the patient and to determine the safety and effectiveness of their treatment.

Protocols (and many other documents produced as part of a trial) are controlled documents, i.e., they are version numbered and dated.

Everyone involved in the trial at ICR-CTSU and at the participating hospitals have to follow the protocol. This ensures all the patients taking part are treated in the same way.

2.2.1 The focus group

In order to help with the setting up and planning of a clinical trial a focus group can be run in order to get input from patients and members of the public. This is very helpful in order to ensure that studies are planned in the best way for patients and barriers to patients taking part are highlighted and resolved where possible. Our focus group will be run as a single 2 hour session with approximately 6-8 patient or public members in the group and 3-4 ICR-CTSU staff. There will be two main themes of the focus group. Firstly, we will discuss the concept of using electronic patient reported outcomes rather than paper versions, the potential benefits and the potential barriers to use. We will then talk in more detail about our plans to run a study within our clinical trials looking at how well patients respond to using electronic patient reported outcomes. We will ask questions on your views on how we have designed the study and whether it could be improved for our patients.

2.2.2 Ethical Review

Before a clinical trial can take place it must be submitted for ethical review. In the UK, the Health Research Authority (HRA) exists to protect the rights, safety, dignity and well-being of research participants. This is achieved by a review of research taking place by a National Research Ethics Service (NRES) Research Ethics Committee. The protocol for any trial cannot be changed without the approval of the ethics committee.

More information about clinical trials can be found on Cancer Research UK's website: http://www.cancerresearchuk.org/about-cancer/find-a-clinical-trial/how-clinical-trials-are-planned-and-organised

2.3 How are clinical trials managed?

Clinical trials are managed by a team of researchers from the planning stage right through to the closure of the trial. Those involved include:

**The Sponsor**	The trial **sponsor** is the individual, company, institution or organisation which is legally responsible for ensuring that there are proper arrangements in place to plan and manage a clinical trial. For trials initiated and managed by ICR-CTSU the ICR usually accepts the sponsorship responsibilities under the UK Clinical Trials Regulations. (Note: The sponsor does not have to be the **funder** of the research. Trials may be funded by charities such as Cancer Research UK, or from pharmaceutical companies, or a combination of several organisations).
**Chief Investigator (CI)**	The Chief Investigator leads the trial and has overall responsibility for the design, conduct and reporting of the trial. In cancer clinical trials the Chief Investigator will be a registered doctor with a strong interest and expert knowledge in the area of research of the trial.
**Principal Investigator (PI)**	If the trial involves more than one hospital, the Principal Investigator is the person at each hospital responsible for the day-to-day running of the clinical trial. (Note: The Chief Investigator can also act as the Principal Investigator for his/her hospital.)

**ICR-CTSU Scientific Lead**	Clinical trials managed by the ICR-CTSU are overseen by an ICR-CTSU Scientific Lead who works closely with the Chief Investigator to ensure that the trial is **scientifically sound** and **managed appropriately**.

The diagram below shows the role of each of the trial team at ICR-CTSU.

**Scientific Lead**	Works closely with the CI to ensure that the trial is scientifically sound and managed appropriately.
**Statistician**	Responsible for working out how many people need to take part in a trial and how the results should be analysed to make sure we can reach a reliable conclusion. During the trial, the statistician monitors the quality of the data collected and produces reports for the Independent Data Monitoring Committee or IDMC. When a trial is finished, the statistician analyses all the data collected in order to work out the results of the trial.
**Clinical Trials Programme Manager (CTPM)**	Working under the direction of the Scientific Lead and ICR-CTSU Operations Director, CTPMs are each responsible for overseeing a component of the trials portfolio, ensuring trials are initiated and delivered effectively.<br>The CTPM is responsible for the development and initiation of new trials and for overseeing the trial team, ensuring staff are suitably trained and supported. In addition, CTPMs provide scientific or operational expertise and leadership within a defined area of research or trial conduct.
**Trial Manager**	Responsible for managing the trial on a day-to-day basis. They are the person you are likely to have most contact with.
**Data Manager**	Responsible for the collection of data, which is the information collected about each person who takes part in the trial. They also assist the Trial Manager in the day-to-day running of the trial.
**Trial Administrator**	Provides administrative support during the development and running of the trial.
**Database Programmer**	Designs and maintains the database/s for the trial.

Further information on the ICR-CTSU can be found at the following locations:
Website: www.icr.ac.uk/our-research/our-research-centres/clinical-trials-and-statistics-unit
Twitter: @ICR_CTSU

2.4 Will I be reimbursed for my expenses?

You will be reimbursed for your time taken to attend and contribute to our focus group at a rate of £25/hour. It is currently expected that the focus group will take place as a videoconference.

3 Any questions?

If you have any questions after reading this information sheet, please get in touch with the SPRUCE team.
